# Reviewers acknowledgement

**DOI:** 10.1002/2211-5463.13937

**Published:** 2024-12-02

The editors of *FEBS Open Bio* would like to thank all those who have given their time and expertise to review articles submitted for publication in Volume 14. Although *FEBS Open Bio* does not seek to judge the importance of submissions, our reviewers carefully scrutinize the experimental design and results of all papers, and challenge authors about their conclusions.

The names of these reviewers are listed below; we apologize if we have inadvertently omitted anyone.

Alexandra Araújo

Jörg Kleeff

Niamh Lynam‐Lennon

Mohammad Aarif Siddiqui

Ashraf N. Abdalla

Rita Abranches

Mariana Acuña

Ruchi Agrawal

Rodrigo Aguiar

Javeed Ahmad

Sachiko Akashi‐Takamura

Yaaser Q. Almulaiky

Cestmir Altaner

Marcelo Alves Pinto

Franca Anglani

Munehito Arai

Sandra Armstrong

Anoop Arunagiri

Avraham Ashkenazi

Deborah L. Baines

Aria Baniahmad

Christian Barbato

Rebecca Barnes

Francis A. Barr

Kaustuv Basu

Zsófia Bata

Howard Baylis

Susanne Bechstedt

Thomas Becker

Iraldo Bello‐Rivero

Alexandre Benedetto

Vitor Bernardes Pinheiro

David Q. Beversdorf

Prasenjit Bhaumik

Sofia‐Iris Bibli

Ricardo Biondi

Eric Blair

Jef Boeke

István Bojti

Stephanie Marie Bozonet

Piotr Bragoszewski

Claudio Brancolini

Stefania Brocca

Laura Brunnthaler

Timothy Bugg

Peter Burkovics

C. Joaquin Caceres

Christine Cavazza

Jobichen Chacko

Dibakar Chakrabarty

Joydeep Chakraborty

Sohini Chakraborty

Etienne Challet

Shih‐Ching Chao

Huaiyong Chen

Qun Chen

Wanlin Chen

Chun‐Jung Chen

Zhe Chi

Jong‐Il Choi

Pooi Yin Chung

Carmen Clapp

Rosemary Clyne

Anne Conibear

Valeria Consoli

Anne Cooke

Fasseli Coulibaly

Xavier Coumoul

Marie Couturier

Nancy Craig

Guillaume Croville

Colm Cunningham

Fiona Cuskin

Boris Cvek

Sakda Daduang

Baoying Dai

Xavier Daura

Peter L. Davies

Caitlin Davis

Iñaki de Diego Martinez

Luitzen de Jong

Juan Vladimir de la Rosa Medina

Ermelinda De Meo

Caio de Oliveira Gorgulho Silva

Danya Dean

Christophe Decroos

Jeroen Den Hertog

Sven Dennerlein

Jayne Dennis

Vincenzo Desiderio

Antimo Di Maro

Cecilia Diaz Oreiro

Victoria Diedrich

Richard Dixon

Chaoqing Dong

Eyal Dor‐On

Jiten Doshi

Senouwa Dossou

Matthew Drew

Joanna Dulińska‐Litewka

Nicolas Dumaz

Katarzyna Patrycja Dzik

Jason Ear

Adrienne Edkins

Sigrun Eick

Andrey Elchaninov

Sherien M. El‐Daly

Toshiya Endo

Ferenc Erdődi

Catherine Etchebest

Abdul Samath Ethayathulla

Chukwuebuka Eze

Isabel Fabregat

Anna C. Fagre

Camille Faure

Roman Fedorov

M. Rohan Fernando

Lucas Ferreri

Eric Allen First

Benjamín Florán

Judith H. Ford

Bernardo Franco

Bryan Grieg Fry

Rao Fu

Ko Fujimori

Mikako Fujita

Satoshi Fukuchi

Atsunori Fukuhara

Fun Man Fung

Vasil Galat

Oxana V. Galzitskaya

Feng Gao

José A. Gavira

James H. Geiger

Alison K. Gillingham

Mario Gimona

Rafael L. Giner Arroyo

Paola Giussani

Nicholas Glykos

Geoffrey N. Gobert

Nady Golestaneh

Georgina Gomez

Saúl Gómez‐Manzo

F. Xavier Gomis‐Ruth

Yoichi Gondo

Shivansh Goyal

Sebastian Greiss

Thomas Grewal

Christian Gruber

Zhenbo Han

Yuichiro Hara

Takeshi Harayama

Joshua M. Hare

Edward N. Harris

Takeshi Hase

Armagan Hayirli

Zhao He

Els Henckaerts

Jonathan Hibshman

Scott W. Hiebert

Mark A. Hink

Rita Hirmondó

Kyle L. Hoehn

Hermann‐Georg Holzhütter

Wanjin Hong

Naoto Hori

Gabriel Hornink

Irena Horwacik

Aaron Hoskins

Susan Ellen Howlet

Shijun Hu

Wonmuk Hwang

Lorena Itati Ibañez

Michael Ibba

Salvador Iborra

Julia Iermak

Kazuhiko Igarashi

Alberto Álvaro Iglesias

Jin Inokuchi

Yoshitaka Isaka

Alexander V. Ivanov

Mohamad Aman Jairajpuri

Jyoti Jaiswal

Sirpa Jalkanen

Dick B. Janssen

Jill L. Johnson

Robbie P. Joosten

Blagojce Jovcevski

Antonija Jurak Begonja

Sebastian Kalamajski

Ashley Kalinski

Mikhail S. Karbyshev

Kazuo Katoh

Maria Kawalec

Sophie Kellaway

Megan Keniry

Bilon Khambu

Alena Khmelinskaia

Seyun Kim

Raymond Kim

Wanil Kim

Yoko Kimura

Philip D. King

Chandra Kishore

Svend Kjaer

Anna Klintsova

Sungjin Ko

Krista Kobylianskii

Masaaki Komatsu

Kulsum Kondiah

Nont Kosaisawe

Bankala Krishnarjuna

Endre Kristof

Kirill Kryukov

Binod Kumar

Ashwini Kumar Ray

Ursula Kummer

Markus Künzler

Samvid Kurlekar

Nikita Kuznetsov

Suresh L. M.

Heather Michelle Lamb

Ola Larsson

Corinne Ida Lasmezas

Jörn Lausen

Elena Lavdovskaia

David S. Leake

Janne Lebeck

Jun Hee Lee

Hyung Ho Lee

Katie Lee

Hsin‐Chen Lee

Hyemin Lee

Jade Leiba

Alexander Leitner

Gaetano Leto

Elena Levantini

Agnieszka Lewinska

Lanlan Li

Jian‐Xin Li

Qizhou Lian

Carla Lima

Jun Liu

Randall Loaiza

Jean‐Marc A. Lobaccaro

Juan Luis López Cánovas

Peter Low

Brandon Lucke‐Wold

Qingjun Ma

Peter Macheroux

Madathiparambil Gopalakrishnan Madanan

Sergio Madrigal‐Carballo

Megan M. Mahoney

Francesco Malatesta

Luigi Manni

Margherita Maranesi

Alvaro Marín‐Hernández

Jonathan Martinez‐Fabregas

Eduardo Martinez‐Hernandez

Teresa Martins

Pedro Martins

Hisao Masai

Dzmitry Matsiukevich

Bernard Maybury

Purabi Mazumdar

Liam Mcdonnell

Heather Bennett Miller

Daniela Miricescu

Aditya Mittal

Ammar Mohammed Al‐Farga

Ofelia Mora

David Moreau

Ryuichi Morishita

Misao Moriya

Charlotte Muir

Raphael Munoz

Ganji Purnachandra Nagaraju

Gergely Nándor Nagy

Nikolett Nagy

Istvan Nagy

Amrinder Nain

Yuji Nakayama

Vigneshwaran Namasivayam

Heinz Peter Nasheuer

Aritro Nath

Sangeeta Nath

Emily Nordmann

Kinga Nyiri

Dominik Oberthuer

Sekyung Oh

Judit Oláh

Vincent Olieric

Baldo Oliva

Michael Olson

Jasminka Omerovic

Eugene Boon Beng Ong

Chris C. Oostenbrink

Sandi Orlić

Natalia Ortiz Chaves

Christiane Ott

Martin Ott

Menno J. Oudhoff

Ali Burak Ozkaya

Petar Ozretić

Nigel Page

Subash Chandra C. Pakhrin

Mark Pallen

Minggui Pan

Nazareno Paolocci

Gianpaolo Papaccio

Jinseok Park

Venkatachalam Parvathi

Manisha Patil

Zeljko Pavkovic

Evgeny V. Pavlov

Nick Peake

Joel Pearson

Nieves Peltzer

Tianqing Peng

Graziano Pesole

Ophry Pines

Sergej Pirkmajer

Sandra Postić

Hemant Kumar Prajapati

Robert Price

Miguel Prudencio

Ellie Putz

Daniel Max Raben

Simon Rabkin

Gergely Attila Rácz

Steve W. Ragsdale

Muhammad Farooq Rai

Amrita Rai

Elena Rainero

Pablo Ranea‐Robles

Sarina Ravens

P. Hemachandra Reddy

Dieter P. Reinhardt

Waleed M. Renno

John Patrick Richard

Jan Riemer

Daniel J. Rigden

Jennifer Rinker

Nirmal Robinson

Martha Robles‐Flores

Josias Rodrigues

Amaia Rodríguez

Helge G. Roider

Alexander Romanishin

Gergely Rona

Jorge Luis Rosas‐Trigueros

Ryan D. Ross

Eitan Rubin

Sara Rubio Sanchez

Shin‐Ichi Sakakibara

Md Samsuzzaman

Prasanna Kumar Santhekadur

Luis Adriano Santod Do Nascimento

Webster Santos

Pierre Santucci

Liel Sapir

Akira Sato

Takahiro Sato

Claude Aage Sauter

Maximilian Scheer

Bohdan Schneider

Karisa C. Schreck

Clara Schroeder

Gerrit Schut

Graham Scott

Barbara Seliger

Marian T. Sepulveda‐Orengo

Diane C. Shakes

Yingjie Shao

Ashu Sharma

Rohit Sharma

Kuang Shen

Tsuyoshi Shirai

Sarah C. Shuck

Roy L. Silverstein

Arjun Singh

Kalkena Sivanesam

Marek Skrzypski

Boris Slobodin

Jim T. Smith

Colin Smith

Rory L. Smoot

Maria Solesio

Iwen Song

Carlos Sorzano

R. Sotelo‐Mundo

Eric Soupene

Erdi Sozen

Sree Rama Chaitanya Sridhara

Piyarat Srisawang

Chloe Stanton

Colin W. Steele

Ioannis Stefanidis

Diana Stojanovski

Ramon Sun

Xueqin Sherine Sun

Adam Szafran

Katarzyna Szkudelska

Carlos Tabraue

Yuichiro Takagi

Marwa Tantawy

Manuel João Tavares Mendes Costa

Chuin Hau Teo

Suman S. Thakur

Pierre Therizols

Kristina Thiel

Anne Tierney

Gabriele Toietta

Tsukasa Tominari

Peter J. Tonge

Renata Torres Da Costa

Masashi Toyoda

Rogelio Treviño‐Rangel

Hiroshi Tsutsumi

Izabela Tuleta

Benjamin Turk

Emel Ulupınar

Tirth Uprety

Miriam Valera‐Alberni

Sakari Vanharanta

Máté Varga

David Vargas Diaz

Silvia Vega Rubin De Celis

Adrian Velazquez‐Campoy

Jorge Verdín

Alexandre Vetcher

Ly Villo

Emma Vincent

Susanne Voelkel

Nora Vögtle

William Wan

Zeneng Wang

Yasuharu Watanabe

Helen Watson

Fiona E. Watt

Ian Webb

Stefan Weger

Lai Wei

Andreas Weigert

Anna Wendt

Felix Wensveen

Nathaniel West

Timothy Whitehead

Angelique Whitehurst

Nils Wiedemann

Dominic Winter

Nadine Wiper‐Bergeron

Darren R. Wood

Louwrance P. Wright

Xue Xiao

Kang Xu

Ruidong Xue

Manoj Yadav

Fábio Seiti Yamada Yoshikawa

Renhong Yan

Beimeng Yang

Michelle Khai Khun Yap

Burak Yazgan

Hironori Yoshino

Michael Zahn

Daniel Zajic

Antonella Zannetti

Markella Zannikou

Bingsen Zhang

Yang Zhou

Guangbiao Zhou

Bo Zhou

Xinzhou Zhu

Yedomon Ange Bovys Zoclanclounon

Ibrahim Zúñiga‐Chaves

